# Genome Sequence of the Autotrophic Acetogen *Clostridium magnum* DSM 2767

**DOI:** 10.1128/genomeA.00464-16

**Published:** 2016-06-09

**Authors:** Ronny Uhlig, Anja Poehlein, Ralf-Jörg Fischer, Rolf Daniel, Hubert Bahl

**Affiliations:** a Institut für Biowissenschaften, Abteilung Mikrobiologie, Universität Rostock, Rostock, Germany; b Genomic and Applied Microbiology & Göttingen Genomics Laboratory, Georg-August University Göttingen, Göttingen, Germany

## Abstract

Here we report the draft genome sequence (6.6 Mbp) of the type strain *Clostridium magnum*, an acetogen with two operons coding for two separate Rnf complexes. *C. magnum* grows on a broad range of organic substrates and converts CO_2_ and H_2_ to acetate using the Wood-Ljungdahl pathway.

## GENOME ANNOUNCEMENT

*Clostridium magnum* DSM 2767^T^ belongs to the group of Gram-positive bacteria with Gram-negative staining. It is an anaerobic, rod-shaped, spore-forming, motile bacterium, which was enriched from pasteurized freshwater sediment with 2,3 butanediol as the sole carbon and energy source by Schink in 1984 (1). In 1991, Bomar et al. (2) showed that *C. magnum* grows with H_2_/CO_2_, formate, or methanol as the substrate when 0.025% (wt/vol) yeast extract was provided in the growth medium. 2,3 Butanediol, acetoin, fructose, glucose, sucrose, xylose, malate, and citrate can serve as the substrates and are completely converted to acetate (1).

Genomic DNA of *C. magnum* DSM 2767 was isolated with the Tissue genomic DNA purification mini prep kit (Genaxxon, Ulm, Germany). Sequencing was done by a combined approach of single-molecule real-time (SMRT) and Illumina sequencing technologies according to the manufacturer’s protocols. Genome sequencing was performed with a PacBio *RSII* (Pacific Biosciences, Menlo Park, CA) using P6 chemistry and an Illumina MiSeq using reagent kit v3 (2 × 300 bp). The *de novo* assembly using the RS_HGAP_Assembly.3” protocol included in SMRT Portal version 2.3.0 and 49,222 postfiltered reads (average read length of 12,759 bp) resulted in 21 contigs with an average coverage of 71.34-fold. The obtained Illumina reads (1,727,812) were quality filtered using Trimmomatic version 0.32 (3) and mapped onto the contigs with Bowtie2 version 2.0.6 (4) to improve the quality of the final sequence. The genome of *C. magnum* consists of a circular chromosome (6,600,766 bp) with an overall G+C content of 32.11%. Automatic gene prediction and identification of rRNA and tRNA genes was performed using the software tool Prokka (5). The genome contains 71 rRNA genes, 125 tRNA genes, 4,392 protein-encoding genes with predicted functions, and 1,773 genes coding for hypothetical proteins.

With a size of 6.6 Mbp, the genome of *C. magnum* is so far the largest sequenced genome of all acetogens, followed by *C. scatologenes* (5.75 Mbp, CP009933), *C. drakei* (5.64 Mbp, JIBU00000000) and *C. carboxidivorans* (5.63 Mbp). The genes encoding enzymes of the Wood-Ljungdahl pathway revealed the same arrangement as that in all other acetogenic bacteria of the genus *Clostridium* (6). Interestingly, *C. magnum* is the first acetogen with a complete additional set of Rnf complex formation-related genes. Moreover, three more genes coding for RnfC-like proteins were found next to genes that are known to be involved in microcompartment formation as well as ethanol, ethanolamine, and 1,2 propanediol degradation (7). The genome analysis furthermore revealed genes encoding proteins for the fixation of molecular nitrogen, sporulation and formation of granulose.

### Nucleotide sequence accession numbers.

This whole-genome shotgun project has been deposited at the DDBJ/EMBL/GenBank database under the accession no. LWAE00000000. The version described in this paper is version LWAE01000000.
